# Supplementary material for: Tolmetin Sodium Fast Dissolving Tablets for Rheumatoid Arthritis Treatment: Preparation and Optimization Using Box-Behnken Design and Response Surface Methodology
Source: Pharmaceutics. 2022 Apr 18;14(4):880. doi: 10.3390/pharmaceutics14040880 (PMC9027483; doi:10.3390/pharmaceutics14040880)
Supplement: Supplementary file 1 [file pharmaceutics-14-00880-s001.zip › pharmaceutics-1663941-supplementary.pdf]

Supplementary data

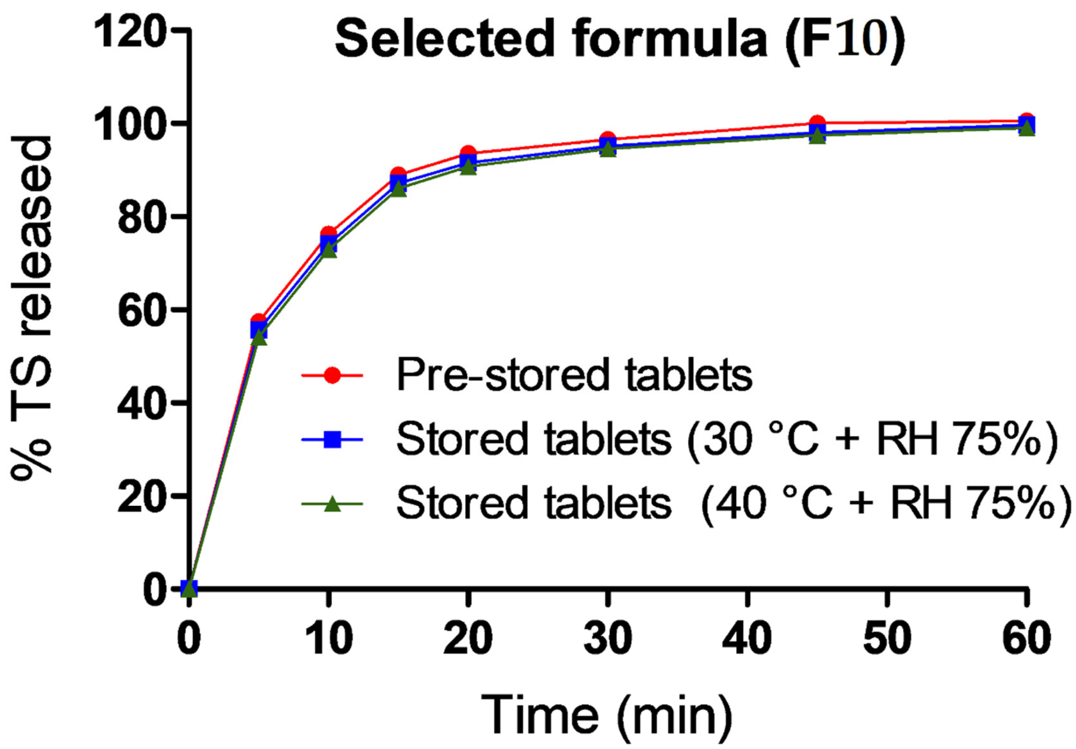

Figure S1. Release profiles of TLM from TLM-FDTs (F11) after storage at 30 and 40 ^{o}C + RH 75% for three months compared with the corresponding recently prepared tablets.

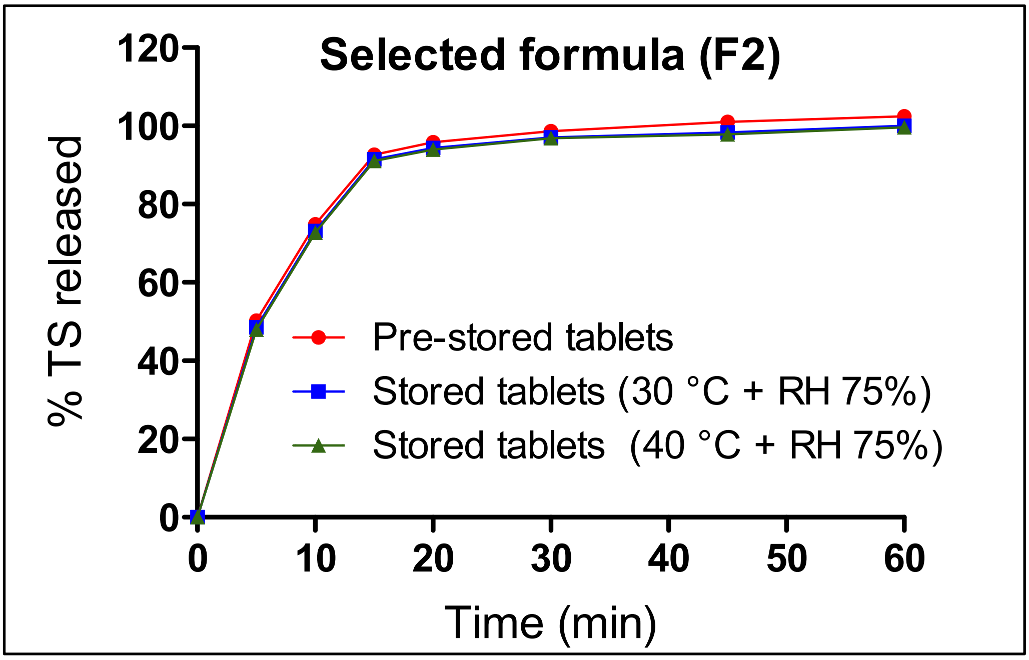

Figures S2. Release profiles of TLM from TLM-FDTs (F2) after storage at 30 and 40 ^{o}C +RH 75% for three months compared with the corresponding recently prepared tablets.
